# A guide for self-help guides: best practice implementation

**DOI:** 10.1080/16506073.2024.2369637

**Published:** 2024-06-20

**Authors:** Roz Shafran, Sarah J. Egan, Madelaine de Valle, Emily Davey, Per Carlbring, Cathy Creswell, Tracey D. Wade

**Affiliations:** a Great Ormond Street Institute of Child Health, University College London, London, UK; b enAble Institute, Faculty of Health Sciences, Curtin University, Perth, Australia; c Discipline of Psychology, School of Population Health, Curtin University, Perth, Australia; d Flinders Institute for Mental Health and Wellbeing, College of Education, Psychology and Social Work, Flinders University, Adelaide, Australia; e Department of Psychology, Stockholm University, Stockholm, Sweden; f Departments of Experimental Psychology and Psychiatry, University of Oxford, Oxford, UK

**Keywords:** Low intensity, guided self-help, evidence-based, cognitive behaviour therapy, stepped care, therapist drift

## Abstract

Guided self-help is an evidence-based intervention used globally. Self-help is a fundamental part of the stepped care model of mental health services that enables the efficient use of limited resources. Despite its importance, there is little information defining the role of the guide and the key competences required. In this context, the guide is defined as the person who facilitates and supports the use self-help materials. This article sets out the role of the guide in guided self-help. It considers practical issues such as the importance of engagement to motivate clients for early change, personalising the intervention, structuring sessions, how best to use routine outcome monitoring and supervision requirements. Key competences are proposed, including generic competences to build the relationship as well as specific competences such as being able to clearly convey the role of the guide to clients. Guides should be prepared for “self-help drift”, a concept akin to therapist drift in more traditional therapies. Knowing how to identify mental health problems, use supervision and manage risk and comorbidity are all key requirements for guides. The paper concludes by calling for increased recognition and value of the role of the guide within mental health services.

## Introduction

Guided self-help interventions have been shown to be effective for a range of mental health difficulties including depression, anxiety, eating disorders, perfectionism, pain, and psychosis (Cuijpers et al., 2020; Davey et al., 2023; Galloway et al., 2022; Liegl et al., 2016; Pauley et al., 2021; Scott et al., 2015; Spanhel et al., 2021). Such guidance is intrinsic to many forms of self-help interventions including internet-delivered guided self-help and guided bibliotherapy (e.g. Carlbring et al., 2011) and has been shown to improve outcomes in self-help treatments with the amount of guidance being correlated with the benefits patients achieve (Johansson & Andersson, 2012) They are a central part of the delivery of mental health services across the globe that can be used in low and middle-income countries as well as their wealthier counterparts (e.g. Tol et al., 2020). Guided self-help interventions are delivered in a range of formats, individually and groups, digitally and analogue, and, realistically, have considerable potential to close the demand-supply gap for mental health interventions. Internet-based CBT often uses guides and, in such cases, can be considered a form of guided self-help although some forms of internet-based CBT are more akin to therapist-delivered interventions whereby the therapist is leading the intervention and uses the materials on the internet to support the therapy and save time (e.g. Clark et al., 2023).

Guided self-help treatments are effective interventions for adults as well as young people (Bennett et al., 2019; McKinnon et al., 2018) and equivalent to face-to-face individual treatment for adults with depression (Cuijpers et al., 2019) as well as psychiatric and somatic disorders (Hedman‐Lagerlöf et al., 2023). Drawing from long-term follow-up data, guided internet-delivered self-help interventions not only demonstrate significant short-term benefits but also show enduring effects, contributing to substantial symptom improvement over extended periods (Andersson et al., 2018). The interventions are underpinned by a strong body of mental health science not just demonstrating their efficacy in randomised controlled trials (e.g. Cuijpers et al., 2019), and their effectiveness in real-world clinical settings (Vangrunderbeek et al., 2022), but also by data examining the mechanism of action (e.g. Lakin et al., 2023).

Despite the importance of guided self-help, there does not appear to be any easily available, published information defining the role of the guide. The guide is akin to a facilitator, helping the person with the mental health difficulty (or their caregiver in the case of parent-delivered guided self-help) to adhere to the intervention with fidelity, to personalise the intervention and to maintain motivation by providing a mixture of support and accountability. The lack of best practice guidance for guides may partly be due to the challenges posed by the wide-ranging communication methods in which the intervention is delivered; guides can engage with clients face-to-face, over the phone, through text messages, or within a virtual reality environment. It may also be due to the wide variety of settings that utilise guided self-help including mental health services, community settings, schools, the workplace and charities. In some settings, such as charities that primarily provide informal support, the boundaries between generic support and specific, formal guided self-help interventions can become blurred. However, guided self-interventions are typically structured, “low intensity” interventions (often cognitive-behavioural in nature) with a clear agenda, content and homework setting that have been evaluated (or are being evaluated) in research trials (Shafran et al., 2021). Guided self-help based on other therapeutic modalities also tends to be structured (e.g. Wade et al., 2022) and has been evaluated with promising results (Mechler et al., 2022). In clinical settings, such as mental health services, qualified clinicians may find it difficult to maintain a clear distinction between acting as a facilitator of self-help and taking on the more extensive role of a therapist. However, it is critical that they are able to distinguish guided self-help from brief therapy, which is more akin to a condensed face-to-face intervention, requires a clinical practice qualification and is less reliant on written materials (Shafran et al., 2021).

An additional challenge in establishing clear and precise best practice recommendations is the diversity of the workforce delivering the guidance. In many settings, there are currently no requirements for guides to have a clinical practice qualification. Such a requirement would prohibit the scalability of guided self-help and limit its ability to close the demand-supply gap. The guided self-help workforce may include assistant psychologists, healthcare support workers, wellbeing practitioners, non-specialist psychology graduates, social workers and charity representatives, as well as peer support workers, experts by experience, and carers supporting their loved ones. Qualified clinicians may provide supervision and can deliver the intervention, but many guides are relatively inexperienced, making it necessary to articulate the role of the guide in general and provide the key principles underlying the provision of guidance to optimise effectiveness. Such a diverse workforce can make it difficult to find the correct language to describe this role with the terms “facilitator” or “practitioner” or ”coach” often used interchangeably with “guide”. We have opted to use the term “guide” to reflect the diversity of the workforce in recognition that while some individuals supporting the delivery of guided self-help interventions will be practitioners with formal mental health qualifications, others will not.

The aim of this paper is to articulate best practice for guides across settings and intervention methods and is based on the collective expertise of the authors having conducted multiple research studies involving guided self-help and many years of experience in delivering self-help interventions in multiple formats.

## Guide role

The guide’s role is to support, encourage and empower the client/caregiver to engage with the intervention to make impactful, lasting change. In contrast to traditional cognitive-behaviour therapy, where typically the therapist would teach the skill in the session for the client to continue to apply beyond the session, in guided self-help clients/caregivers will have received the intervention content (e.g. a chapter or module or handout materials) prior to the session. Specifically, the tasks as a guide are to assist the client (or their caregiver in the case of parent or caregiver-delivered guided self-help) to:

- Engage with the intervention content
- Understand and reflect on the intervention content
- Personalise the intervention content to consider how it can best be applied given the individual circumstances
- Problem-solve any challenges in applying the intervention content
- Identify and use the client/caregiver’s own strengths to help them
- Help empower the client/caregiver to make change
- Help maintain momentum
- Celebrate wins (this includes the courage to try something new even if it doesn’t go to plan) such that hope and motivation to persist in change is enhanced

The guide can provide clarification and correct misunderstandings but should stay focused on helping clients to implement the skills learned, to motivate and encourage clients to do the homework by practising the skills learned, and to address problems and barriers. It is essential that the guide is motivational and encouraging (Berg et al., 2022). To be able to fulfil the role, the guide needs to be very familiar with the intervention content, with an interesting aspect of using self-help treatments being that they also aid student therapists to understand the treatment material better. The guide should be fully aware of every element of the intervention so that they can personalise content and facilitate the balance between fidelity and flexibility (Kendall et al., 2023). Such familiarity with the content allows for a fluidity of sessions and helps engagement which in turn can build the momentum for early change which is so important for effective outcomes (Beard & Delgadillo, 2019). Ideally, the guide should have foundational knowledge of the mental health disorder with which they are working, the clinical features, common treatments, and the research on interventions and recovery. The guide should not be introducing the material for the first time to the client/caregiver in the session. Nor should the guide provide advice, for example on general life issues or problems. The goal is for the client or parents/caregivers to feel empowered to use their own skills and judgement in using the intervention rather than feeling reliant on the guide to solve their problems. Instead of providing advice, the guide should be a sounding board for parents/caregivers to think about how they might apply strategies (e.g. “That sounds like a tricky situation, what are your ideas for how to handle it?” or “How have you handled challenges like this in the past?”, ”Could you apply anything from that situation to this one?” or “Is there anything you read from the therapy materials over the last week that may give you some ideas on this?”). Familiarity with the content means that the guide can also direct them to parts of the program that might be relevant (e.g. “It sounds like there’s a negative prediction getting in your way—maybe it would be helpful to re-read Module X on behavioural experiments this week and see if you can set up an experiment to test that idea”).

Within talking therapies services in the UK, the guide’s role is distinguished from that of a traditional therapist in a number of ways. The guide, in the form of a Psychological Wellbeing Practitioner is described predominantly as a “coach” – supporting, enabling and motivating the patient to use evidence-based published manuals, self-help guides or other CBT self-help materials to work towards recovery (PWP Best Practice Guide, 2015). In such services, the guide typically works with people with mild to moderate mental health difficulties in recognition that they do not have the skills to support those with serious and enduring mental health problems or people with high levels of risk. In contrast, ”high intensity” therapy is provided by a trained therapist with a recognised core profession (e.g. clinical psychologist) who can manage such complexity and risk and typically delivers a range of psychological interventions over more sessions; such ”high intensity” standard therapy is not driven predominantly by the materials but instead by a combination of clinical judgement, research evidence and patient preference.

## A ‘cheat sheet’

For each guided session of the intervention, it is helpful for the guide to have summaries of the content of the intervention and the homework exercises. If they are not freely available, then it is recommended that guides make their own summary and also create a brief checklist for each session—ticking off each part to ensure all the parts are covered, though they need not be covered always in the same order, to allow for flexibility. An example is shown in Table 1.

**Table 1.** Checklist for each session.

Session Item	Key Points	Tick
Check weekly questionnaire.	Check whether they completed the weekly questionnaire	
	If not, have them complete it in-session before proceeding	
Risk Check-In	Ask about levels of risk	
FIRST SESSION ONLY Introduce yourself and your role if you did not do so in the assessment.	Your name and qualifications	
	Explain the role of the guide	
	Discuss confidentiality and its limits	
Check module completion	Ask whether they completed Module 1 (i.e. read the content, completed the within-module exercises)	
	Help to problem-solve any challenges to completion of Module 1	
Collaborative agenda-setting.	Ask if there are any questions or challenges that they would like to discuss with you (you will cover these next). *Make note of their agenda item(s):*	
	Explain what you’d like to discuss today:	
Parent/caregiver agenda item(s).		
Module content	Topic 1	
	Topic 2	
	Topic 3	
Homework	Help to problem-solve any barriers to completing the homework	
	Discuss outcomes if homework is already completed	
Confirm details of and preparation for next session.	Agree on date and time of when they will do the next module and when you will have the next session	
	Explain what they should do before the next session: Homework from Module 1 if not done already. Module 2 Weekly questionnaire	

## Agenda

Guides will be following a protocol and applying it flexibly but with fidelity. A key component of the guidance session is to ask the client/caregiver what they would like to cover in the session when setting the agenda. Clients/caregivers should be asked first what they would like to cover, rather than the guide telling them what the guide plans to cover and then simply asking “is that ok?” or “anything you want to add”. The reason to ask clients/caregivers first is to convey the message that the treatment is collaborative, the content is being personalised and that this time is for the client/caregiver to cover what they need to—and the guide will help troubleshoot those difficulties. It may be the case that the client/caregiver does not have anything to add to the agenda because they have not managed to read a new chapter or cover the module content or do the homework. The guide should check on barriers, help the client to use a problem-solving framework to identify and resolve challenges and check motivation to continue, but not proceed with material for the next session or skip the topic. Instead, the guide should encourage the client/caregiver to try again. Reviewing the homework and scores on the session-by-session weekly measures together will help the guide identify what should be on the agenda and address the challenges encountered.

## Session by session measurement

Many guided self-help interventions have regular progress monitoring built in. This is because of the strong data showing that such progress monitoring improves retention and outcomes in mental health interventions (de Jong et al., 2021; Delgadillo et al., 2018). Guides should be familiar with the measures that are used and should be aware that ensuring completion of the weekly questionnaires is a central part of the role as the guide. Guides should encourage clients/caregivers to complete the weekly questionnaires prior to the sessions to accurately reflect what has happened in the week and to optimise time spent in the session. If the client/caregiver has not completed them, it is important to spend time on this in the session with a clear rationale that such measurement is a key component of effective treatment. A visual display of progress (e.g. a line graph depicting changes in scores over measurement points) can easily be made using software such as Excel (see supplementary material). Such a display can help the client and guide to easily reflect on progress in sessions, identify barriers to change and guide the direction of the intervention (Law & Wolpert, 2014). Given that 6% of patients undergoing psychological treatments, including guided cognitive—behavioural therapy, may experience deterioration during their treatment, visual displays can help with the early detection of such deterioration and provide opportunities for intervention (Rozental et al., 2018).

## Session structure, timing and administration

In contrast to traditional psychological therapy where sessions are usually around 50 minutes, guided self-help sessions are usually around 20–30 minutes in duration. Ideally in each session, the client or caregiver should complete the module or chapter and have attempted the homework **prior** to attending the guided self-help session and completing the weekly questionnaires. The guide will often have administrative tasks prior to the session to make sure the client/caregiver can complete the module prior to the session, such as sending the weekly measures (or link to the weekly measures) as well as the module content. It is good to discuss expectations of guided self-help at the initial assessment session as well as timings of sending materials. For example, it may be that it is best for the guide to send the materials for the following week immediately after the guidance session so that the client has the most time and opportunity to work through it and identify any difficulties. It may also be a good idea to discuss with the client/caregiver having a regular time slot for them to work through the content. For example, it may be agreed for the client/caregiver to receive the questionnaires and module content and handouts on a Friday immediately after the guidance session, schedule time in their own diary to go through it over the weekend and return any materials on the Wednesday. If the next session with the guide is on the following Friday, then the guide has time to review the materials prior to the session which can help plan the session appropriately. The therapist and client should collaboratively decide the particular day of the week to check in on treatment progress.

To reduce administration time and thereby maximise the cost-effectiveness of this approach, guides may benefit from developing template emails for routine use. For example, they might develop separate templates to send following each guidance session that contains the measure(s) to complete before the next session (or a link to these measures), the content for the next module/section, an area to insert the agreed-on time and place for the subsequent session, and the upcoming homework exercises. These can be flexibly adapted as needed to include additional elements, such as words of encouragement after encountering difficulties or a reminder to complete outstanding homework. These email templates could also be provided to guides by services or research teams, to increase uniformity of the approach across guides (see supplementary material for examples of email templates).

There will be variation across settings with regard to the implementation of guided self-help. For example, in Sweden, the initiation of a guidance session generally depends on the patient submitting their homework. Following the submission, the guide provides feedback on the completed assignment. A frequently encountered issue arises when patients do not submit their homework, resulting in a failure to trigger the scheduled guiding session. To address this, it is a common practice for the guide to proactively reach out to the patient in cases where no homework has been submitted. This ensures consistent weekly communication between the guide and the patient, although the focus and content of the session may vary depending on the circumstances.

## Competences

There are generic competences that are essential for optimal guidance. Such competences include a positive, respectful, non-judgemental attitude, the ability to empathise, to be interested in the client and to summarise information presented (UCL Competency Maps, 2007). All of these will help with engagement and building the relationship, which is critical to the successful implementation of the materials and outcome, regardless of whether the guidance is being provided face-to-face, over the telephone or digitally in written form. Other generic competences include being able to establish relationships with relevant professionals. Competences for cognitive behaviour therapy guided self-help include providing the client with a rationale for the self-help model in an encouraging and realistic manner, helping the client understand that the main role of the guide is to facilitate the use of self-help material(s) and conveying to the client the collaborative nature of a self-help intervention (UCL Competency Maps, 2007). Formulation and goal-setting skills are required for many guided self-help interventions (Cromarty & Gallagher, 2023), with goal-based outcomes commonly used for weekly measurement, particularly for young people (Law & Jacob, 2013). A standardised measure of the assessment and treatment competencies to deliver low-intensity CBT may be helpful for guides to reference, particularly during their training (Kellett et al., 2021).

## Troubleshooting lack of engagement

In a short session with significant content to cover, it is sometimes difficult to provide the support and encouragement needed to help the client/caregiver make full use of the intervention, especially when the client/caregiver does not appear to be fully engaged. However, it is at those times that the client/caregiver is most in need of empowerment. Lack of engagement can be used as an opportunity to explore and problem-solve the obstacle, to change without being critical, and to reflect on alternative strategies. Maintaining a positive stance and highlighting what has gone well in addition to what has not can be helpful, particularly for clients/caregivers for whom a negative outlook and hopelessness are part of the difficulties. Encouraging the idea that there isn’t a “right” and “wrong” way to think, feel or behave but there are different options at different times can help with self-criticism and dichotomous thinking that can be an obstacle to change. At times, it is tempting for the guide to increase flexibility with regard to availability for appointments or to delay deadlines, but it is important to remain mindful of the potential negative impacts of providing too much deadline flexibility, as this can inadvertently lead to poorer outcomes, as highlighted by Paxling et al. (2013).

## Supervision

Regular supervision from a qualified, senior clinician is essential. Ideally, the supervisor themselves will have clarity and expertise in the delivery of guided self-help to ensure that they fully understand the role of the guide. It is important that the supervisor is themselves clear that the primary purpose of the supervision is to make sure the guide is not stepping out of their role, to support the guide in helping the client/caregiver engage with the materials, to maintain a focus on outcomes and to ensure that risk and any deterioration is being suitably monitored and handled. The supervisor can also help the guide monitor any other negative effects of the intervention (Rozental et al., 2018). It is extremely helpful for audio and video-recordings of the sessions to be made for such purposes, although it is our impression that this is unfortunately relatively rare in routine clinical practice. Audio-recording telephone sessions may require specialist equipment, but this is not expensive, and sessions that take place via video-conferencing can typically be easily recorded. Concerns over data protection, governance, and uncertainty about what to do with the recordings in the longer term may play a role in the reluctance to record sessions and so it is critical that services provide information on their policies and procedures to guides at the outset.

It is recommended that guides use a supervision sheet such as the one shown in Table 2 that incorporates the key components of supervision including which audio or visual clip will be played and the visual record of weekly progress monitoring to help identify risk and monitor deterioration and other negative effects.

**Table 2.** Example form for supervision.

Name:
Date:
**Questions for supervision** What do you want to get out of this session? What audio (or video) clip would it be most helpful to focus on today? Make a note of the section you want to play and question you want to ask. Any questions of risk of harm to self or others should take priority
**Client progress** List all your cases, session number currently on and how many planned, summary of changes on outcome measures (graphed if possible) and any challenges the client is having with using the guided self-help materials
**Cases to discuss** These should include any cases in which there has not been improvement in the outcome measures or there is deterioration or other negative effects
**Content of supervision** Main points discussed
**Action Plan** What are you going to do while remaining clearly within your role as guide?
**Date, Time and Place of next supervision**

Engaging in role-plays is useful for training and supervision and exploring different scenarios but can be resisted by guides due to fear of evaluation. Supervision competences for low-intensity interventions have been clearly set out (Roth & Pilling, 2015) and, as applied to guided self-help, the competences include the ability to:

- Identify and discuss any misconceptions that the guide may hold regarding the rationale for, and application of, guided self-help
- Help the guide draw on knowledge of the rationale for guided self-help interventions, and on the evidence base for their use
- Support the guide in assessing suitability for guided self-help
- Support the guide’s delivery of guided self-help
- Support the guide in use of routine outcome monitoring
- Support decisions about the appropriateness of interventions and, in conjunction with the guide, determine when it might be appropriate to consider onward referral.

Services will differ in the amount of supervision offered to guides but Talking Therapies in the UK suggest that qualified low intensity practitioners should receive a minimum of 1 hour of clinical case management supervision per week by an experienced therapist as well as 1 hour of clinical skills supervision (either individually or in a group format) every two weeks (NHS England, 2023). Further information as to the nature of supervision for such services can be found in the Talking Therapies manual (NHS England, 2023). Other services will have different guidelines. For example, for parent-delivered guided self-help comprising a welcome module (0), 6 core modules and a follow-up module (Creswell et al., 2017), the guide is encouraged to bring clients to supervision following Modules 1, 3, and 6 unless guides pre-emptively need support thinking about “SMART” goals and step plans, in which case, clients can be brought to supervision before these modules. Guides are also encouraged to bring clients to supervision prior to or following the follow-up appointment if further treatment and/or referrals to other services are warrantedand/or following Module 4 or Module 5 if the young person has not made at least 3 points progress towards any of their “SMART” goals or if they are less than halfway towards achieving their main goal.

## Risk and safeguarding

Identifying and addressing risk of harm to the client or others is clearly of utmost importance but it is also challenging. Guided self-help sessions often start with a risk check-in. Services or research study leads must ensure that guides are fully aware of relevant protocols, policies and procedures with regard to identifying and managing risk. In addition, guides would benefit from being aware of national clinical guidelines on identifying risk such as those produced by the National Institute of Health and Care Excellence (National Institute of Health and Care Excellence, 2022) and the World Health Organisation (World Health Organisation, 2021).

## Co-occurring difficulties

It can be particularly challenging for a guide to facilitate the implementation of a self-help intervention in the context of co-occurring difficulties. Typically, guided self-help interventions are for specific difficulties such as eating difficulties, anxiety, depression or behaviour. However, most clients will have multiple problems, and the likelihood is that guides will not be intimately familiar with treatment protocols for every single mental health disorder due to their training, which is by necessity limited in scope (see Wade et al., 2023). However, individually tailored guided self-help interventions have shown demonstrated effectiveness and cost-efficiency even in the presence of co-occurring difficulties (Nordgren et al., 2014). Bringing a description of the symptoms to supervision is the first step, and ongoing training is likely to be required to help guides recognise symptoms of a range of common comorbidities (e.g. eating difficulties and anxiety, obsessive compulsive disorder and depression, psychosis and trauma). Identifying symptoms of potential comorbid conditions and bringing them to supervision will help establish whether it is appropriate to continue with the guided self-help protocol or whether a different intervention may be required and how best to monitor comorbidities using standardised measures. The decision about how best to address comorbidities should be made in the context of the literature on co-occurring difficulties that, although preliminary, suggests that staying focused on one mental health problem can provide benefits in other areas (e.g. Gibbons & DeRubeis, 2008; Mahdi et al., 2019). Transdiagnostic interventions delivered in a guided self-help format may also be an alternative option (Dalgleish et al., 2020).

The summary for guides delivering self-help for a specific mental health disorder in the context of co-occurring mental health disorders is:

- Keep focused—mental health comorbidity does not necessarily mean you need to deviate from the plan
- Continue to monitor the co-occurring symptoms to establish if additional intervention required (potentially at the end of the focused treatment)
- Be explicit with clients with co-occurring difficulties that targeting one area can lead to benefits in others (both for transdiagnostic processes such as perfectionism but also for disorder-specific interventions), and that the plan is to keep focused but continue to monitor other difficulties to determine if additional interventions may be needed.

## Self-help ‘drift’

The concept of therapist drift is well described and refers to clinicians failing to deliver the optimum evidence-based treatment despite having the necessary tools (Waller, 2009). We hypothesise that guide drift and self-help drift are also likely to occur for very similar reasons, such as knowledge, attitude, anxiety, experience, age, theoretical orientation, critical thinking, personality traits and cultural competency (Speers et al., 2022). The strategies recommended for therapists could equally well be applied to guides and the clients/caregivers who are using the self-help intervention in terms of using cognitive behavioural techniques to address malleable factors such as negative beliefs about a specific intervention (Waller & Turner, 2016). Additionally, guides should be aware of the types of interventions that they and the clients/caregivers are most likely to avoid utilising a priori, typically behaviour-based strategies involving exposure (Whiteside et al., 2016), and consider strategies such as using checklists to help ensure fidelity to the intervention.

## When guided self-help is not enough

Guides should have realistic expectations of outcomes. Some clients may choose not to undergo self-help treatments but instead opt to wait for a more intensive, traditional form of therapy if it is available. Additionally, although outcomes with guided self-help are excellent overall, there will be many clients/caregivers who need additional support and research suggests that there are some client variables that increase the odds of “stepping up” from a self-help to a more intensive intervention such as pre-treatment severity and client preference (Nicholas et al., 2019). Services will undoubtedly have their own criteria for when clients can be “stepped up” to receive such additional interventions, typically in the form of traditional therapy delivered by a qualified, specialist clinician or to alternative options such as group treatments. Services need to ensure that guides are fully aware of relevant service criteria in relation to how to provide clients/caregivers with available treatment options as soon as the need is identified.

## Disseminating guidance

We are mindful that individual research groups and clinical services have extensive, specific written guidance to support the implementation of their specific guided self-help programme that could be usefully applied elsewhere. We encourage services and researchers to publish their guidance alongside the intervention to facilitate optimal implementation.

## Concluding comments

We wrote this paper due to the surprising gap in the literature for guides who are tasked with providing guided self-help and wish to learn about the requirements and responsibilities of this role. Understanding the unique role of the guide is the first step to acquiring the competences required to empower the client to change, provide encouragement, personalise the intervention, structure and organise sessions and use progress monitoring to maximum effect. There may be differences in how guides are utilised within services, and session structures may vary but fundamentally this is an essential role across global mental health systems that warrants appropriate investment of research, clinical and training resources.

## Supplementary Material

Supplemental Material
